# Increased PD-1 expression in livers associated with PD-1-antibody-induced hepatotoxicity

**DOI:** 10.1186/s12865-025-00682-y

**Published:** 2025-01-23

**Authors:** Miro Saarela, Essi Parviainen, Ana Lleo, Luca di Tommaso, Hanna Raunio, Krista Kankaanranta, Katri Vuopala, Aino Rönkä, Sini Nurmenniemi, Raija Kallio, Arja Jukkola, Katri S. Selander

**Affiliations:** 1 Department of Oncology and Hematology, Oulu University Hospital, Oulu, Finland; 2 Translational Medicine Research Unit, University of Oulu, Oulu, Finland; 3 Department of Biomedical Sciences, Humanitas University, Pieve Emanuele, Milan, Italy; 4 Internal Medicine and Hepatology Unit, Department of Gastroenterology, IRCCS, Humanitas Research Hospital, Rozzano, Milan Italy; 5 Department of Pathology, IRCCS Humanitas Research Hospital, Rozzano, Milan Italy; 6 Department of Oncology, Tampere University Hospital, Tampere, Finland; 7 Tampere Cancer Center, Faculty of Medicine and Health Technology, Tampere University, Tampere, Finland; 8 Department of Pathology, Lapland Central Hospital, Rovaniemi, Finland; 9 Oncology, Institute of Clinical Medicine, University of Eastern Finland, Kuopio, Finland; 10 Cancer Center, Kuopio University Hospital, Kuopio, Finland

**Keywords:** Checkpoint inhibitors, Adverse event, Lymphocyte infiltration

## Abstract

Vanishing bile duct syndrome (VBDS) is a serious drug induced liver injury characterized by chronic cholestasis and loss of intrahepatic bile ducts. VBDS has been reported also following checkpoint inhibitor treatment. We compared CD3 + , CD4 + , CD8 + , CD20 + , CD57 + , PD-1 + and PD-L1 + lymphocyte infiltrates in liver biopsies of patients that encountered VBDS (*n* = 2) or hepatotoxicity (*n* = 3) after pembrolizumab (*n* = 4) or nivolumab (*n* = 1) treatment with samples from normal liver (*n* = 10), non-alcohol steatohepatitis (NASH, *n* = 10), primary biliary cholangitis (PBC, *n* = 10) or pembrolizumab-treated patients without adverse events (*n* = 2). Notably, none of the cancer patients had primary nor metastatic liver tumors. We also studied direct growth effects of pembrolizumab on primary human intrahepatic biliary epithelial cells (HIBEpiC) in vitro. Liver sections of all checkpoint inhibitor- treated patients exhibited significantly higher CD3 + infiltration than normal livers, and significantly higher PD-L1 + , CD4 + and CD8 + infiltration, than other groups. PD-1 + infiltration was significantly increased in livers of patients with severe hepatic adverse event. CD57 + infiltration was similar in normal livers, NASH- and PBC groups, but highly increased in the checkpoint inhibitor-treated patients. Immune cell infiltrates were similar between NASH and normal livers. PBC samples had significantly higher CD3 + , CD4 + , CD8 + and CD20 + infiltrates than normal livers. HIBEpiC express PD-L1 but pembrolizumab did not affect their viability in vitro. Our findings suggest that VBDS is not due to direct cytotoxicity of checkpoint inhibitors and that the immunological attack against livers induced by these drugs is different from other cholestatic liver conditions.

Biological insight: Checkpoint inhibitors upregulate PD-1 and PD-L1, as well as cytotoxic CD57 + cells in the non-cancerous liver tissues and this may be associated with checkpoint inhibitor-induced hepatotoxicity.

**Supplementary Information:**

The online version contains supplementary material available at 10.1186/s12865-025-00682-y.

## Introduction

Checkpoint inhibitors are monoclonal antibodies that target CTLA-4 (ipilimumab), PD-1 (nivolumab, pembrolizumab) or PD-L1 (atezolizumab, durvalumab), on T-cells or cancer cells. These drugs unlock the antitumor efficacy of oncolytic T- cells, enabling immunological destruction of tumors [1]. Their clinical adoption has dramatically changed the course of various metastatic cancers [2]. These drugs are also being increasingly studied and used in earlier cancer stages [3, 4].

Checkpoint inhibitors may also induce toxic side effects in healthy tissues, which are usually successfully managed with immunosuppressants [5, 6]. Some side effects may, however, occur rapidly and can be resistant to immunosuppressants [3, 4, 6–8].

Vanishing bile duct syndrome (VBDS) is an example of a rare but serious side effect associated with these drugs [3, 4, 7, 9]. To gain more understanding of VBDS pathophysiology, we studied immune cell infiltrates in liver biopsies of patients that had either a transient liver injury or fatal or non-fatal VBDS, or no adverse events in response to pembrolizumab or nivolumab and compared them with those from non-alcohol steatohepatitis (NASH), primary biliary cholangitis (PBC) or normal livers.

## Methods

### Patient samples

We studied liver biopsies of patients, that experienced hepatotoxicity (*n* = 5) or had no adverse effects (*n* = 2) after treatment with pembrolizumab or nivolumab at the Department of Oncology, University Hospital of Oulu, University Hospital of Kuopio, University Hospital of Tampere, Finland or at the IRCCS Humanities Research Hospital, Rozzano, Milan, Italy. According to inclusion criteria, all patients were adults, and none had primary liver tumors or liver metastases. Hepatic viral infections or known contraindications for checkpoint inhibitors were exclusion criteria. Information of the five patients that experienced a serious treatment-induced treatment associated hepatic side effect is given on Table 1. Samples of normal liver, NASH and PBC were obtained from the Biobank Borealis (Oulu, Finland). Such samples were selected based on the diagnosis.

**Table 1:** Characteristics of patients that experienced checkpoint-inhibitor treatment associated hepatotoxicity

	**Patient # 1**	**Patient # 2**	**Patient # 3**	**Patient # 4**	**Patient # 5**
**Cancer**	**Melano**ma	**RCC**^a^	**Melanoma**	**NSCLC**^b^	**Colon cancer**
-Stage	IV	III	III	IV	IV
**Checkpoint- inhibitor**	**Pembrolizumab**	**Pembrolizumab**	**Nivolumab**	**Pembrolizumab**	**Pembrolizumab**
-# of treatments	1	7	12	1	3
**Symptom onset **					
-days after previous anti-PD-1 treatment	1	67	48	20	39
**Immunosuppressants**	**Yes**	**Yes**	**Yes**	**No**	**Yes**
-Steroids	+	+	+	-	+
-Myconophenolate	+	+	+	-	**+**
-Days used prior to liver biopsy	4	43	7	-	10
**Outcome**	**Fatal**	**Survived**	**Survived**	**Survived**	**Survived**
**Highest value:**					
-Bilirubin	655	401	269	752	123
-ASAT	835	405	150	261	
-ALAT	1769	2137	364	818	916
-GGT		1716	411	5148	1744
-ALP		598	466	478	668
-INR	3.5	1.1	1.1		1.0
-NH4	53	66			
Bile ducts vanished	Yes	No	Partial	Yes	No
Cholestasis	Yes	Yes	Yes	Yes	Yes

^a^Renal cell carcinoma

^b^Non-small cell lung carcinoma

### Lymphocyte quantitation

Cut sections of liver biopsies were stained with antibodies against CD3 (NCL-L-CD3-565), CD4 (NCL- L-CD4-368), CD8 (NCL-L-CD8-4B11) all from Novo Castra Leica, CD20 (MO755, clone L26, Dako), CD57 (Natural Killer Cell Marker, Thermo Scientific), PD-1 (ab2587, Abcam) and PDL-1 (E1L3N, Cell Signaling) with standard immunohistochemistry. The stained slides were scanned with Leica Aperio Slide scanner. The images were analyzed by QuPath positive pixel count, by calculating the percentage of immunopositive pixels of all pixels of the section, with the following settings: Downsample factor 2.0, Gaussian sigma 1 and DAB threshold 0.3 [10].

### Cell viability assays

Primary Human Intrahepatic Biliary Epithelial cells (HIBEpiC) were plated on 96-well plates (1000 cells in 100 µl per well) in manufacturer recommended normal growth medium (ScienCell Laboratories) and cultured at 37° C under standard conditions [11]. The next day, vehicle, pembrolizumab or doxorubicin (Selleck Chemicals), as a well-known cancer medication and known inhibitor of cellular growth, were added to the cells [12]. Cellular growth as a function of time was analyzed with MTS-assays as previously described [11].

### Western blotting

Human MDA-MB- 231 breast cancer cells and murine J774 macrophages were obtained from ATCC and cultured as previously described [10, 11]. Peripheral blood mononuclear lymphocytes (donated by healthy volunteers and isolated with Ficoll) were extracted with RIPA buffer (Bio-Rad) and ran on Novex^TM^4-20% Tris-Glycerin Mini Gels (Thermo Fisher), 25 µg denatured protein per lane. The separated proteins were transferred to nitrocellulose membranes, which were incubated with either anti-PD-1 (D4W2J) XB® Rabbit mAb #86,163) or anti PDL-1(E1L3N®, XP® rabbit MAB #13,684) antibodies (diluted at 1:1000), followed with secondary antibody (1:30,000, Anti-rabbit IgG (H + L) (DyLight™ 800 4X PEG Conjugate #5151, all from Cell Signaling Technology). The membranes were scanned with LI-COR Odyssey using fluorescence at 800 nm. For loading control, the membranes were stripped and re-probed with beta-actin loading control monoclonal antibody (BA3R, DyLight 680, Thermo Fisher Scientific, 1:1000) and scanned using fluorescence at 680 nm.

### Statistical analyses

Data is expressed as mean ± S.D. or ± S.E.M., as indicated. Statistical analyses were performed with GraphPad Prism version 7.0 (GraphPad Software Inc., San Diego, CA, U.S.A), using Student’s T-test. P-values smaller than 0.05 were considered statistically significant.

## Results

**Fig. 1:**
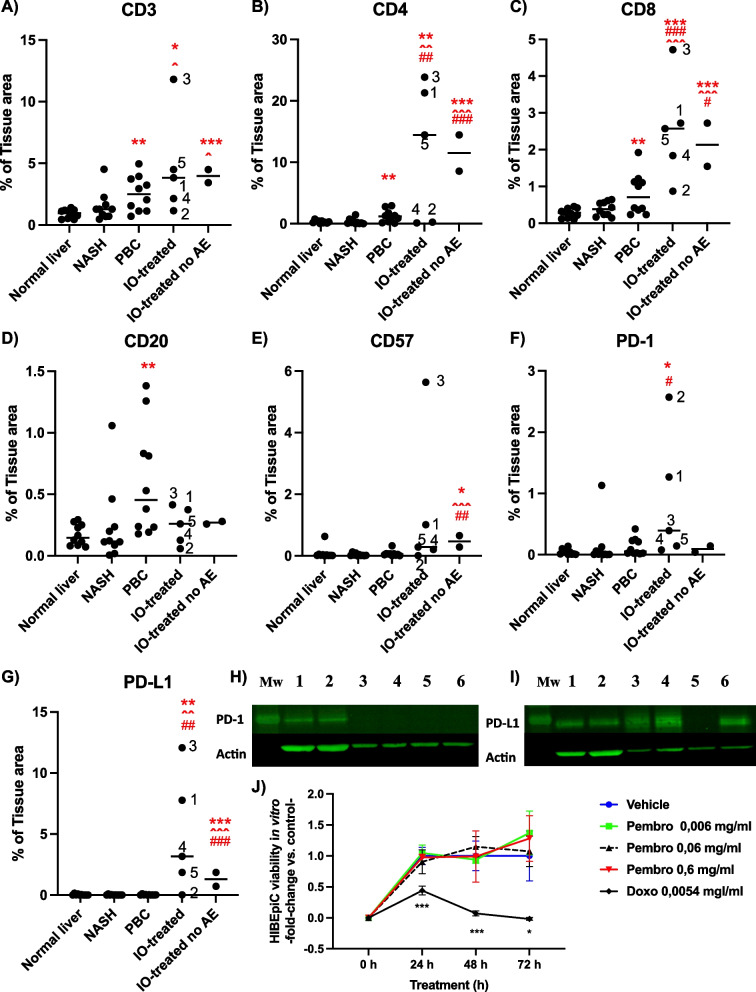
Lymphocyte profiling in liver tissues. **A** CD3 + , **B** CD4 + , **C** CD8 + , **D** CD20 + , **E** CD57 + , **F** PD-1 + and **G** PD-L1 + positive tissue areas, representing corresponding lymphocytes in liver biopsies from normal livers (*n* = 10), NASH (*n* = 10) or anti-PD-1-antibody-treated patients with hepatotoxic (IO-treated, pembrolizumab- or nivolumab-treated patients 1–5, corresponding to the patients listed in Table 1.) or without adverse events (IO-treated no AE) patients (*n* = 2). Individual values for each patient data points are shown, the transverse bars represent the mean value, * *p* < 0.05, ** *p* < 0.01, *** *p* < 0.001 vs. normal livers, ˆ *p* < 0.05, ˆˆ*p* < 0.01 ˆˆˆ*p* < 0.001 vs. NASH, # *p* < 0.05, ## *p* < 0.01, ### *p* < 0.001 vs. PBC. **H** PD-1 and **I** PD-L1 protein expression in samples: 1 – 2) PBMC cells from volunteers, 3 – 4) human MDA-MB-231 breast cancer cells, 5) murine J774 macrophage cells and 6) HIBEpiC cells. B-actin of the same blots are shown to demonstrate protein loading. (Uncropped blots are shown in Supplementary Fig. 1.) **J** HIBEpiC viability as a function of time in the presence of indicated concentrations of pembrolizumab (pembro, corresponding to ~̴ 0.4, 4 or 40 pM), doxorubicin (doxo, corresponding to ~̴ 10 µM) or vehicle (control). Data is expressed as mean ± S.D., (*n* = 5), and presented as fold-change vs. control, * *p* < 0.05, ** *p* < 0.01, *** *p* < 0.001 vs. vehicle

CD3 + tissue areas were significantly higher in the livers of anti-PD-1-antibody- treated patients, as compared with normal and NASH livers (Fig. 1A). Livers from anti-PD-1- antibody- treated patients also exhibited statistically significantly higher CD4 + , CD8 + and PD-L1 + tissue areas, as compared with the other groups (Fig. 1B, C, G). CD20 + areas were similar in the anti- PD-1-antibody- treated and other groups (Fig. 1D). CD57 + stained areas were higher among anti-PD-1-antibody treated patients than in the other groups. This difference reached a statistical significance between the anti-PD-1-antibody-treated patients without adverse events and normal liver, NASH and PBC groups (Fig. 1E). PD-1 + tissue area was statistically significantly higher among the patients that experienced a serious hepatic adverse effect from anti-PD-1-treatment, as compared with normal liver, NASH and PBC groups. Although the distribution of PD-1 + staining was higher in the group that experienced serious hepatic injury, the anti-PD-1-antibody-treated groups did not statistically differ from each other in this regard or for other analyzed lymphocytes either. This may be due to small sample sizes and requires the experiment to be repeated with larger numbers. CD3 + , CD4 + , CD8 + and CD20 + areas were significantly higher in the PBC group than in the normal livers (Fig. 1A-D). No differences were detected between NASH and normal liver groups (Fig. 1A-G).

As demonstrated with Western blotting, HIBEpiC cells express PD-L1, but not PD-1 (Fig. 1H-I). Pembrolizumab, tested at concentrations ranging from tenfold below and above the therapeutic plasma concentrations, did not affect HIBEpiC cell viability in vitro [13]. Doxorubicin was used as a positive control for inhibition of cell viability. As expected, doxorubicin inhibited HIBEpiC cell viability (Fig. 1J).

## Discussion

Vanishing bile duct syndrome is a rare hepatic side effect of various drugs, including checkpoint inhibitors [3, 4, 7–9]. The pathophysiology of this condition, especially after checkpoint inhibitor treatment, is not well understood [8]. There are, however, several publications reporting the development of VBDS as early as after the first checkpoint inhibitor infusion [3, 4, 7, 8]. This was also the case also with one of our own patients. To gain further understanding on this issue, we compared liver immune cell infiltrates of pembrolizumab- or nivolumab- treated patients with or without treatment- associated hepatic adverse effects to those of normal livers, NASH and PBC. The most striking finding here was that PD-L1 expression was significantly higher in the liver sections of anti-PD-1-antibody-treated patients, as compared with other groups. Furthermore, PD-1 + cells were significantly increased among patients that experienced treatment-induced serious hepatic adverse event, as compared with normal livers or PBC. No increase in PD-1 + areas were detected in specimens from patients without such adverse effects. Despite small sample size and the lack of pre-treatment control samples, these findings agree with previous reports describing increased PD-L1 expression in cancer tissues and peripheral blood T-lymphocytes after pembrolizumab- treatment [14, 15]. These findings further suggests that anti- PD-1 antibodies may increase the expression of their own target. As such, this finding would offer a plausible explanation as to why these drugs may induce favorable treatment responses in malignancies even when PD-L1 expression is initially absent. Further investigation is needed to define whether this phenomenon also contributes to the serious hepatic side effects [7]. Although none of our patients had primary or metastatic liver tumors, there was a significant infiltration of CD3 + , CD4 + and CD8 + lymphocytes into the livers with these treatments. Increased inflammation in portal areas, without specification of the lymphocytes, has also been described in previous publications of checkpoint-inhibitor-induced VBDS [7].

Our results lend further proof to an immunological insult as the mechanism for the serious side effects of checkpoint inhibitors. The nature of such insult needs to be better defined as typically, VBDS did not respond to corticosteroids or other immunosuppressants in our patient or in those previously described [3, 4, 7, 8]. These results also further suggest that anti- PD-1- antibody treatment- induced autoimmunity differs from that of other autoimmune liver conditions, such as PBC. This is also in agreement with previous publications [7]. Specifically, the high number of CD57 + cells among half of the anti-PD-1-antibody treated patients with transient liver injury was notable. CD57 expression has been detected in both natural killer (NK) and T- lymphocytes [16]. CD57 expressing lymphocytes are also unable to proliferate and display high cytotoxic potential [16]. Our finding needs, however, to be confirmed in a larger cohort. If true, CD57 could be a potentially novel target to manage serious hepatic side effects of checkpoint inhibitors.

## Conclusions

Our results suggest that severe anti-PD-1- antibody induced liver toxicity is not due to direct cytotoxicity against bile duct epithelial cells, but likely immunologically mediated. These treatments appear to induce immunological cell infiltration also into tissues, that do not contain cancer. Furthermore, anti-PD-1-antibody- induced hepatotoxicity appears to be immunologically different from PBC.

## Supplementary Information

Supplementary Material 1.

## Data Availability

Relevant datasets are available from the corresponding author upon reasonable request.
