# Supplementary material for: Increased PD-1 expression in livers associated with PD-1-antibody-induced hepatotoxicity
Source: BMC Immunol. 2025 Jan 23;26:4. doi: 10.1186/s12865-025-00682-y (PMC11755961; doi:10.1186/s12865-025-00682-y)
Supplement: Supplementary file 1 — Supplementary Material 1. [file 12865_2025_682_MOESM1_ESM.docx]

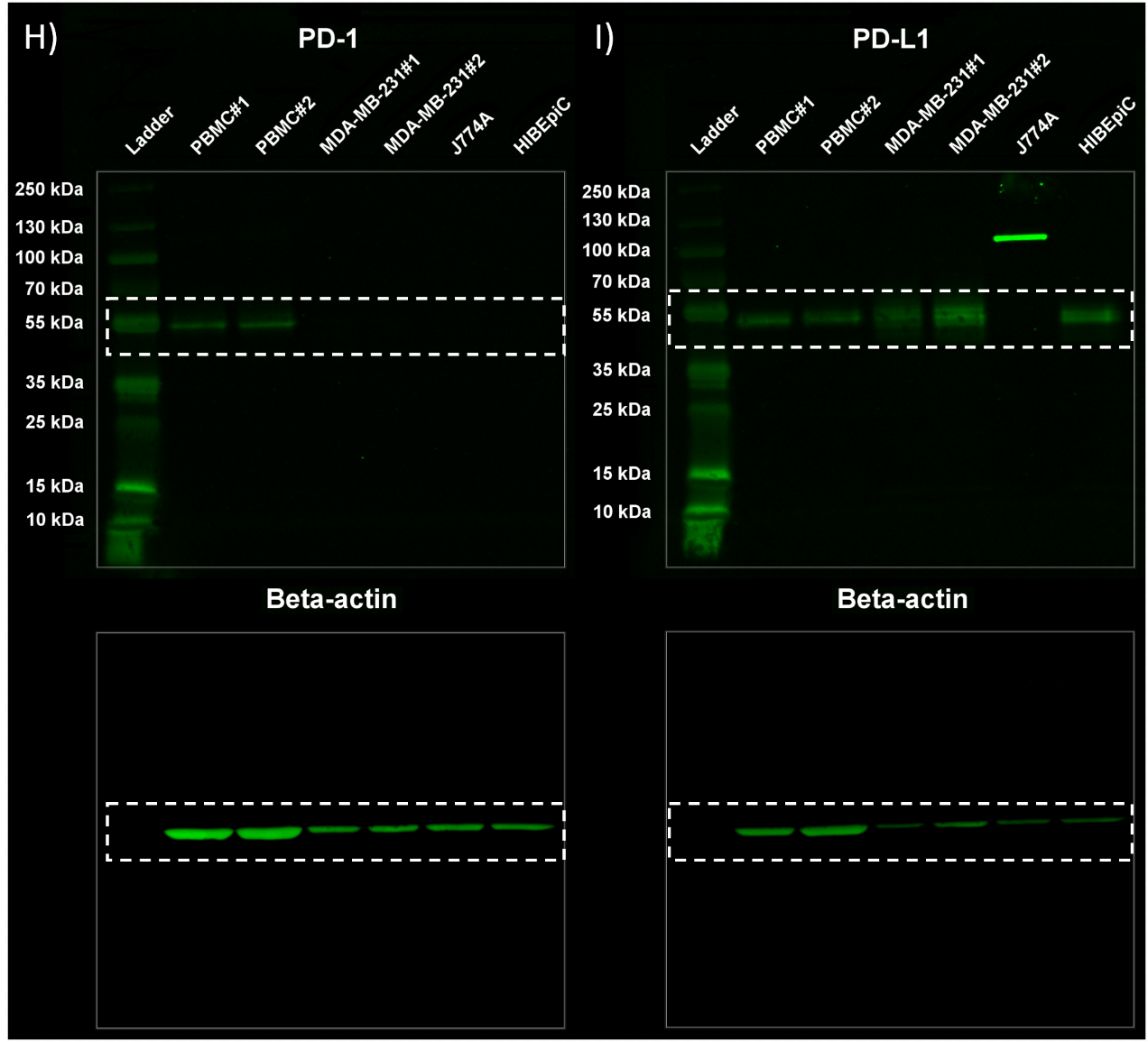


**Supplementary Figure 1.** Uncropped blots for Figure 1. H) PD-1 and I) PD-L1 protein expression in samples: 1 – 2) PBMC cells from volunteers, 3 – 4) human MDA-MB-231 breast cancer cells, 5) murine J774 macrophage cells and 6) HIBEpiC cells. B-actin of the same blots are shown below to demonstrate protein loading.
